# Ultrasound‐Guided Thoracic Paravertebral Block With Liposomal Versus Standard Bupivacaine for Older Patients After Thoracoscopic Surgery

**DOI:** 10.1002/kjm2.70244

**Published:** 2026-06-11

**Authors:** Wen‐Cheng Shi, Ying‐Jie Tang, Yao Wang, Zhong Zheng, Jing He

**Affiliations:** ^1^ Department of Anesthesiology Taicang First People's Hospital Taicang China

**Keywords:** liposomal bupivacaine, opioid‐sparing, postoperative pain, thoracic paravertebral block, video‐assisted thoracoscopic surgery

## Abstract

The study aimed to investigate the effect of thoracic paravertebral block (TPVB) with liposomal bupivacaine (LB) before anesthesia induction on postoperative pain, quality of recovery (QoR), and opioid consumption in older patients after video‐assisted thoracoscopic surgery (VATS). A total of 116 older patients who were scheduled to undergo VATS were allocated into a SB group (TPVB with standard bupivacaine [SB] before anesthesia induction) and a LB group (TPVB with LB before anesthesia induction). The primary outcome was the Numerical Rating Scale (NRS) scores of patients measured at 6, 12, 24, 36, 48, and 72 h postoperatively. The secondary outcomes were score of the QoR‐15 questionnaire at 24 h postoperatively, effective times of patient‐controlled intravenous analgesia (PCIA), cumulative sufentanil consumption, first time to request for analgesia, incidence of adverse events, and incidence of postoperative delirium. The LB group had significantly lower NRS scores at 24 and 36 h postoperatively than those in the SB group. The LB group exhibited significantly higher QoR‐15 scores at 24 h postoperatively, fewer effective times of PCIA, less cumulative sufentanil consumption from 24 to 72 h postoperatively, and longer time to first request for analgesia than the SB group. The two groups did not differ in incidences of adverse events and postoperative delirium. The study provides evidence that TPVB with LB is effective in alleviating postoperative pain at 24 and 36 h postoperatively, improving QoR, and providing opioid savings for older patients after VATS.

## 1 Introduction

Video‐assisted thoracoscopic surgery (VATS) has revolutionized thoracic surgeries and remains the gold standard for lung cancer surgery due to its benefits for patients by minimizing surgical trauma, reducing postoperative pain, and accelerating postoperative recovery [1]. Even with minimal incision, VATS still leads to moderate‐to‐severe acute postoperative pain with limited functional recovery in 12.7% of patients at 24 h postoperatively and 15.6% at 48 h postoperatively [2]. Inadequate postoperative pain control is associated with an increased risk of postoperative pulmonary complications, which is also a risk factor for the development of chronic postoperative surgical pain [3]. Concerning older patients undergoing VATS, perioperative management is still challenging due to physiological debilitation, and they may experience a higher incidence of postoperative complications than younger patient groups [4]. For many anesthesiologists and thoracic surgeons, reduced postoperative pain with early quality of recovery (QoR) remains a significant challenge [5].

Regional analgesia technique is an important component of multi‐modal analgesia that has been widely used for patients undergoing VATS [6]. Thoracic paravertebral block (TPVB) has been recommended as a first‐choice regional anesthesia for VATS by accommodating local anesthetics that can spread into all surrounding spaces including intercostal, interpleural, epidural, and prevertebral spaces, thus offering targeted pain relief with minimal respiratory compromise [7, 8]. At present, prolonging the duration of local anesthetics continues to be significant challenge in TPVB [9]. LB is a multivesicular formulation allowing extended local anesthetic release up to 72 h and providing a mechanistic advantage over standard bupivacaine (SB) [10]. Recently, several studies have investigated TPVB with LB in VATS, reporting a significant pain relief for patients receiving LB compared to those receiving ropivacaine or SB [11, 12]. However, there is limited evidence focusing on postoperative pain relief offered by TPVB with LB for older patients undergoing VATS. The study aimed to investigate the effect of TPVB using LB before anesthesia induction on postoperative pain, QoR, and opioid consumption in older patients after VATS. The researchers hypothesized that TPVB with LB could effectively alleviate postoperative pain, accelerate QoR, and reduce postoperative opioid consumption without adding adverse events in older patients after VATS.

## 2 Methods

### 2.1 Study Participants and Setting

This is a randomized controlled trial study recruiting older patients who were scheduled to undergo VATS for lung cancer between June 2024 and June 2025. The inclusion criteria were: (i) patients pathologically diagnosed as primary lung cancer; (ii) patients undergoing elective unilateral VATS; (iii) patients with the American Society of Anesthesiologists (ASA) physical status classification I–III; and (iv) patients aged ≥ 65 years. The exclusion criteria were: (i) allergy to any of the drugs used in the study; (ii) severe coagulation dysfunction, cirrhosis, renal impairment, severe hypertension, known mental illness; or malignancy other than primary lung cancer; (iii) chronic (persistent or recurrent) pain ≥ 3 months before the study; (iv) the long‐term use of steroids; (v) according to preoperative imaging data, serious intrathoracic adhesion affecting the operation under VATS; or (vi) refusal to provide written informed consent. The elimination criteria were conversion to open thoracotomy or experiencing severe surgical complications during the perioperative period. The study protocol complied with the principles of the Declaration of Helsinki and was approved by the Ethics Committee of our hospital. All participants provided written informed consent.

### 2.2 Sample Size Calculation

The sample size calculation was based on the primary outcome and computed with prior‐power analysis using the G*power software (version 3.1.9.2). The primary outcome was the Numerical Rating Scale (NRS) scores of patients measured at 6, 12, 24, 36, 48, and 72 h postoperatively. A prospective pilot study that had been performed at our institution in March 2024 was used for sample size estimation based on the NRS scores at 24 h postoperatively using Student's *t*‐test. The results showed a significant difference between the NRS score of 2.3 ± 0.9 in the SB group and 1.8 ± 0.5 in the LB group at 24 h postoperatively. The reason why we chose NRS scores at 24 h postoperatively is that LB is reported to prolong the duration of analgesia between 24 and 72 h postoperatively [13]. Based on a two‐tailed test with *α* = 0.05 and power = 0.90, a minimum of 96 participants was required. Considering a 20% attrition rate, the estimated sample size was a minimum of 116 in total (58 per group), thus maintaining robust statistical power to support reliable inference.

### 2.3 Randomization and Blinding

Randomization to the treatment condition was performed by an independent researcher outside the trial. The total sample size was estimated to be 116 (58 for the SB group and 58 for the LB group), and thus 116 random numbers were generated using Microsoft Excel. Each random number corresponding to one participant was written on a separate card and then placed into sealed, opaque envelopes. To ensure allocation concealment, the envelope remained sealed until opened by the TPVB operator on the day of surgery after patient enrollment. The attending anesthesiologist was not blinded to the trial grouping due to the nature of the study. The assessor, unaware of group assignments, entered all collected data into an Excel spreadsheet. Data were coded and stripped of identifying information before analysis. Group allocation was only revealed after the statistical analyses were completed.

### 2.4 Ultrasound‐Guided TPVB Procedures

After admission to the operating room, each patient was given standard monitoring and positioned laterally for TPVB before general anesthesia. All TPVB procedures were performed by two senior anesthesiologists (Y.‐J.T and Y.W.). The TPVB was performed at the paravertebral space 2 cm from the midline of the spine and the upper and lower adjacent paravertebral space where the planned surgical incision was located and guided by an ultrasound machine (SonoScape, Shenzhen, China). The convex array probe (5–10 MHz low‐frequency) was perpendicular to the intraplane approach of the spine to visualize the paravertebral space. After skin disinfection, a 21‐G nerve puncture needle was inserted deep into the triangular space formed by the pleural parietal layer, intercostal lining, and tip of the transverse process under ultrasound guidance. Upon confirming the absence of blood or cerebrospinal fluid via aspiration, 20 mL SB or LB was administered under real‐time ultrasound visualization, with 6.6 mL in the paravertebral space where the incision will be located and 6.6 mL in the two adjacent paravertebral spaces. SB (75 mg; Zhaohui Pharmaceutical, Shanghai, China) was diluted with normal saline to a final volume of 20 mL and LB (266 mg; Jiangsu Hengrui Medicine Co Ltd., Jiangsu, China) was diluted to a final volume of 20 mL.

### 2.5 Anesthesia and Analgesia Management

The general anesthesia was induced with 0.02 mg/kg midazolam, target‐controlled infusion of propofol at a plasma target concentration of 4.0 μg/mL, 0.5 μg/kg sufentanil, and 0.6 mg/kg rocuronium. Upon the train‐of‐four ratio (TOFR) reaching 0, a double‐lumen endotracheal tube was inserted under video laryngoscopy guidance. To maintain general anesthesia, propofol was administered at a rate of 4–10 mg/kg/h and remifentanil was administered at a rate of 0.1–0.3 μg/kg/min, with bispectral index (BIS) maintained at 40–60.

After surgery, all patients received the patient‐controlled intravenous analgesia (PCIA) and the instructions for the PCIA pump were performed by the nurse blinded to the group assignments. The PCIA consisted of 100 μg sufentanil and 16 mg ondansetron hydrochloride diluted to 100 mL with 0.9% normal saline and was configured as no background infusion, a bolus amount of 2 mL, a locking time of 15 min, and maximum hourly dose of 10 mL. The duration of PCIA use was from post‐anesthesia care unit discharge until 72 h after surgery. The pain degree of patients was evaluated using the NRS, and 3 mg of morphine was intramuscularly administered as a rescue analgesic when the patients reported intolerable pain or their pain was scored ≥ 4 on the NRS.

### 2.6 Outcome Measures

The primary outcome was the score of NRS at 6, 12, 24, 36, 48, and 72 h after surgery. The NRS is the most common pain assessment tool in sample of older individuals with pain, which is standardized on a scale of 0 to 10 suggesting pain degree from no pain to “worst imaginable pain” [14]. The secondary outcomes were the score of QoR‐15 questionnaire at 24 h after surgery, cumulative sufentanil consumption within 72 h postoperatively, the effective times of PCIA, the time of first request for analgesia, the remedial dose of analgesia consumption, the incidence of adverse events, the incidence of postoperative delirium at 72 h postoperatively, and the length of postoperative hospital stay. The QoR‐15 is a validated patient‐centered tool that assesses the quality of postoperative recovery, including five items about physical comfort, two items about physical independence, two items about psychological support, two items about emotional state, and two items about pain [15]. Each item was scored on a 10‐point scale, with a total score ranging from 0 to 150. Higher QoR‐15 scores indicate greater quality of postoperative recovery. The incidence of adverse events including hypotension, bradycardia, nausea and vomiting, and dizziness was recorded. The severity of adverse events was classified as mild or severe grade. Mild adverse events included postoperative nausea and vomiting (mild: nausea only; moderate: nausea and one vomiting episode; severe: ≥ 3 vomiting episodes or complications requiring intervention), fever, dizziness, somnolence, urinary retention, hypoxemia, hypertension, hypotension, postoperative atelectasis, and pneumonia. Severe adverse events included myocardial infarction, stroke, severe arrhythmia, pulmonary embolism, acute respiratory distress syndrome, and heart failure. Mild adverse events were managed with supportive interventions (e.g., rest or nonprescription medications) and promptly documented in case report forms. Severe adverse events were immediately reported to the institutional ethics committee according to protocol.

### 2.7 Statistical Analysis

Collected continuous data were checked for normal distribution by the Shapiro–Wilk test. When normally distributed, continuous data were described as a mean value with a standard deviation (SD) or 95% confidence interval (CI); otherwise, median with interquartile range (IQR, Q1, Q3) supplemented reporting. Data of mean with SD were analyzed by parametric tests (independent *t*‐test) and data of median with IQR by nonparametric tests (Mann–Whitney *U* test). Repeated‐measures NRS pain score data were analyzed using two‐way repeated‐measures ANOVA. The model included a group‐time interaction term to assess differential effects over time, and Bonferroni correction was applied for multiple comparisons. Intent‐to‐treat analyses with multiple imputations for missing data were used on pre‐ and post‐test data. Categorical data are reported as numbers with percentages (%) and analyzed by using the chi‐squared tests. All statistical tests used a two‐tailed *p* < 0.05 as statistically significant in GraphPad prism, version 8.0 (GraphPad, San Diego, CA, USA).

## 3 Results

### 3.1 Participants Characteristics

**FIGURE 1:**
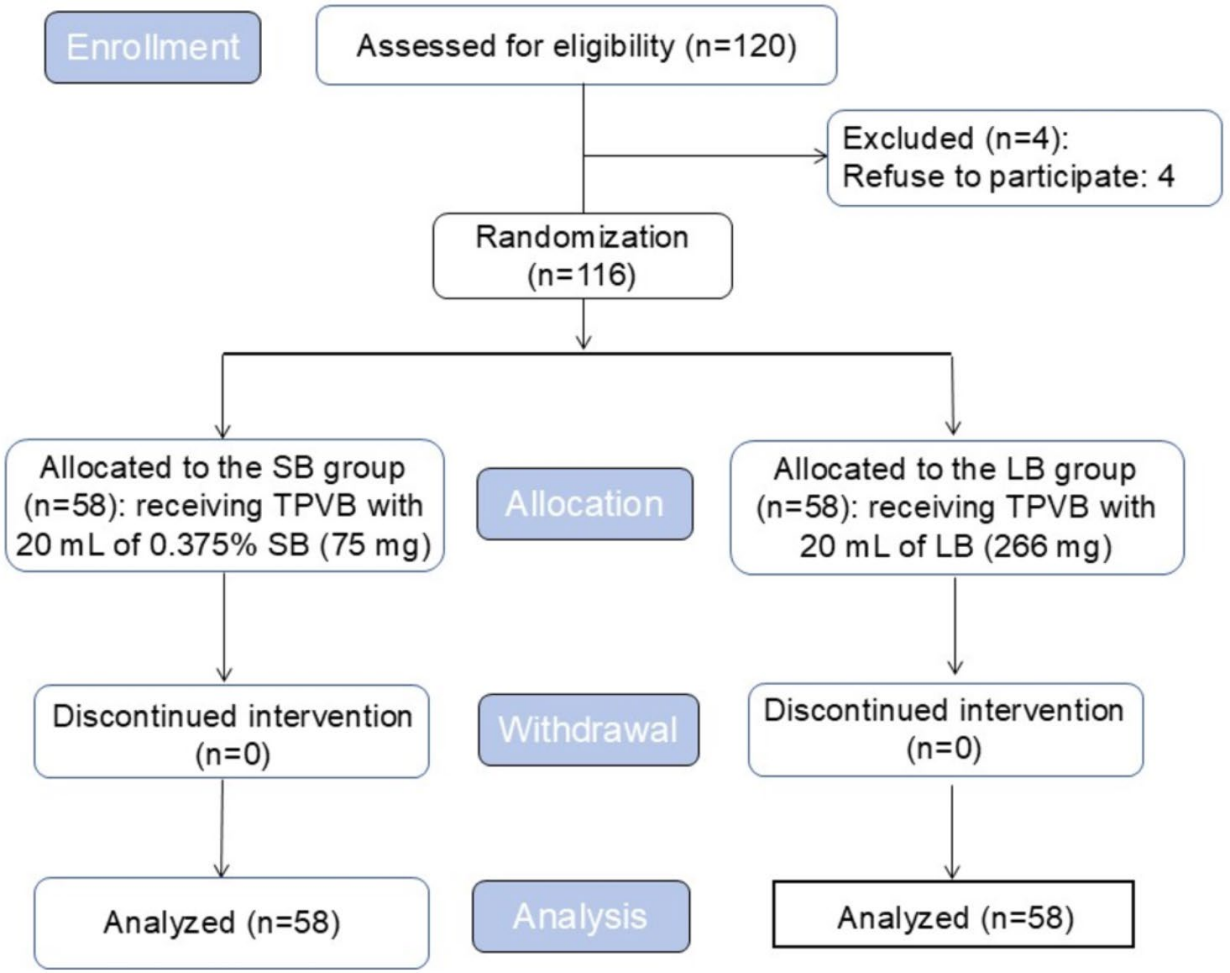
The flowchart of participant recruitment. LB, liposomal bupivacaine; SB, standard bupivacaine; TPVB, thoracic paravertebral block.

During the study period, a total of 120 older patients who were scheduled to undergo elective unilateral VATS were initially selected for this study, and 4 were excluded due to their refusal to participate in the study. The 116 enrolled patients were randomly allocated into SB (*n* = 58) or LB (*n* = 58) groups with 1:1 allocation (Figure 1). Demographic and surgical characteristics of participants (Table 1) did not differ between SB and LB groups.

**TABLE 1:** Demographic and surgical characteristics of participants.

Characteristic	SB (*n* = 58)	LB (*n* = 58)	*p*
Age (years), mean ± SD	73.0 ± 3.8	73.7 ± 4.0	0.308
Sex, *n* (%)			0.710
Male	26 (44.8%)	28 (48.3%)	
Female	32 (55.2%)	30 (51.7%)	
BMI (kg/m^2^), mean ± SD	24.5 ± 3.4	24.9 ± 3.2	0.494
Hypertension (yes), *n* (%)	25 (43.1%)	20 (34.5%)	0.341
Diabetes mellitus (yes), *n* (%)	3 (5.2%)	4 (6.9%)	0.697
ASA classification, *n* (%)			0.243
I	20 (34.5%)	18 (31.0%)	
II	37 (63.8%)	35 (60.4%)	
III	1 (1.7%)	5 (8.6%)	
Surgical type, *n* (%)			0.708
Lobectomy	22 (37.9%)	18 (31.0%)	
Segmentectomy	8 (13.8%)	10 (17.3%)	
Wedge resection	28 (48.3)	30 (51.7%)	
Intraoperative sufentanil (μg), median (Q1, Q3)	30 (25, 30)	30 (25, 30)	1.000
Intraoperative remifentanil (mg), median (Q1, Q3)	0.70 (0.43, 0.86)	0.64 (0.38, 0.89)	0.515
Operation duration (min), mean ± SD	124.0 ± 27.8	121.7 ± 31.7	0.677
Number of blocked dermatomes, median (Q1, Q3)	5 (4, 6)	5 (4, 6)	0.725

*Note:* Data summarized as mean ± SD are analyzed by unpaired *t*‐test. Data summarized as median (Q1, Q3) are analyzed by Mann–Whitney *U* test. Data shown as numbers (percentage) are analyzed by the chi‐square test.

Abbreviations: ASA, American Society of Anesthesiologists; BMI, body mass index; LB, liposomal bupivacaine; SB, standard bupivacaine.

### 3.2 Primary Outcomes

**FIGURE 2:**
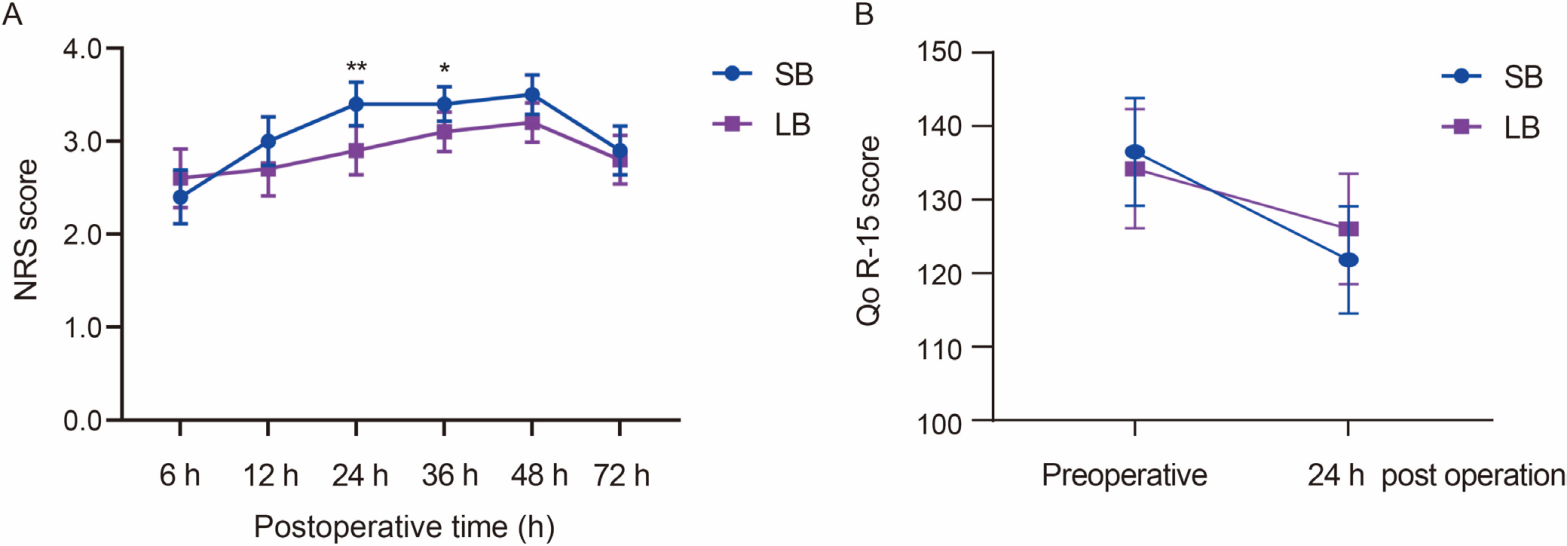
The outcome trajectories over time by group. (A) The NRS scores at predetermined intervals (6, 12, 24, 36, 48, and 72 h postoperatively) between SB and LB groups; data summarized as mean with 95% CI are analyzed by two‐way repeated‐measures ANOVA and Bonferroni correction for multiple comparisons. (B) The QoR‐15 scores at 24 h postoperatively between SB and LB groups; data summarized as mean ± SD are analyzed by unpaired *t*‐test. *p < 0.05, **p < 0.01. LB, liposomal bupivacaine; NRS, Numerical Rating Scale; QoR‐15, quality of recovery‐15; SB, standard bupivacaine.

The NRS scores of patients between the LB group and the SB group were measured at predetermined intervals (6, 12, 24, 36, 48, and 72 h postoperatively). Two‐way repeated‐measures ANOVA revealed an interaction between group and time (*F*_time×group_ = 2.71, *p* = 0.036). The following multiple comparisons (Bonferroni correction) showed that the patients in the LB group had significantly lower NRS scores at 24 and 36 h postoperatively than those in the SB group (*p* = 0.005; *p* = 0.014; Table 2 and Figure 2A). As for other time points postoperatively (6, 12, 48, and 72 h), the NRS scores were similar between SB and LB groups (*p* > 0.05).

**TABLE 2:** NRS scores at 6, 12, 24, 36, 48, and 72 h postoperatively between SB and LB groups.

	SB (*n* = 58)	LB (*n* = 58)	*p*
NRS score, mean (lower‐upper 95% CI)			
Postoperative 6 h	2.4 (2.1–2.7)	2.6 (2.3–2.9)	0.372
Postoperative 12 h	3.0 (2.7–3.3)	2.7 (2.4–3.0)	0.149
Postoperative 24 h	3.4 (3.1–3.6)	2.9 (2.6–3.1)	0.005
Postoperative 36 h	3.4 (3.2–3.7)	3.1 (2.8–3.3)	0.014
Postoperative 48 h	3.5 (3.3–3.7)	3.2 (3.0–3.4)	0.098
Postoperative 72 h	2.9 (2.6–3.1)	2.8 (2.5–3.0)	0.511

*Note:* Two‐way repeated‐measures ANOVA revealed an interaction between group and time and Bonferroni correction was applied for multiple comparisons.

Abbreviations: 95% CI, 95% confidence interval; LB, liposomal bupivacaine; NRS, Numerical Rating Scale; SB, standard bupivacaine.

### 3.3 Secondary Outcomes

**FIGURE 3:**
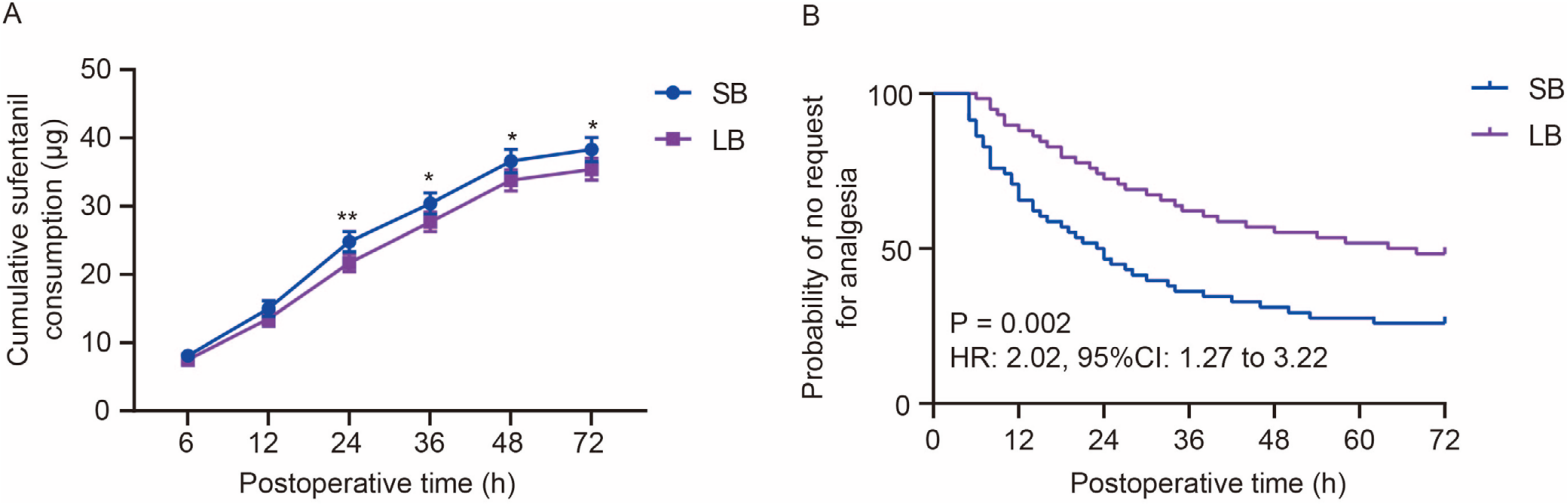
The cumulative sufentanil consumption at each time point within 72 h postoperatively and the survival curve of first time to request for analgesic between SB and LB groups. (A) The cumulative sufentanil consumption; data summarized as mean ± SD are analyzed by unpaired *t*‐test. *p < 0.05, **p < 0.01. (B) The survival curve of first time to request for analgesic. LB, liposomal bupivacaine; SB, standard bupivacaine.

The patients in the LB group had significantly higher QoR‐15 scores at 24 h postoperatively than those in the SB group (*p* = 0.002; Table 3 and Figure 2B). Fewer effective times of PCIA were found in the LB group than in the SB group (*p* = 0.016; Table 3). The cumulative sufentanil consumption from 24 to 72 h postoperatively was notably less in the LB group than that in the SB group (Figure 3A). As for rescue analgesia, the LB group had a longer time to first request for analgesia than the SB group (*p* = 0.002; Figure 3B). The two groups did not differ in the incidence of adverse events, the incidence of postoperative delirium, and the length of postoperative hospital stay (*p* > 0.05; Table 3).

**TABLE 3:** The secondary outcomes between SB and LB groups.

	SB (*n* = 58)	LB (*n* = 58)	*p*
QoR‐15 score, mean ± SD			
Preoperative	136.5 ± 7.3	134.2 ± 8.1	0.118
Postoperative 24 h	121.8 ± 7.2	126.0 ± 7.5	0.002
Effective time of PCIA, mean ± SD	19.2 ± 3.3	17.7 ± 3.0	0.016
Adverse event, *n* (%)	23 (37.9%)	16 (27.6%)	0.169
Hypotension			0.377
No	50	53	
Mild	8	5	
Bradycardia			0.542
No	51	53	
Mild	7	5	
Nausea and vomiting			> 0.999
No	55	55	
Mild	2	1	
Moderate	1	2	
Severe	0	0	
Dizziness			0.464
No	53	55	
Mild	5	3	
Postoperative delirium, *n* (%)	10 (17.2%)	7 (12.1%)	0.431
Length of postoperative hospital stay (days), median (Q1, Q3)	9 (7, 11)	8 (6, 10)	0.202

*Note:* Data summarized as mean ± SD are analyzed by unpaired *t*‐test. Data shown as numbers (percentage) are analyzed by the chi‐square test.

Abbreviations: LB, liposomal bupivacaine; PCIA, patient‐controlled intravenous analgesia; QoR‐15, quality of recovery‐15; SB, standard bupivacaine.

## 4 Discussion

The present study proved the primary hypothesis that use of LB in ultrasound‐guided TPVB provided prolonged postoperative analgesia at 24 and 36 h postoperatively compared to SB for older patients undergoing VATS and secondary hypotheses that this thoracic regional anesthesia also contributed to an improved QoR and exhibited opioid‐sparing effects for these populations.

Earlier work has shown promising implications of LB in postoperative pain control without adding significant adverse events in multiple surgical settings, such as hemorrhoidectomy [16], breast reconstruction [17], total knee arthroplasty [18], and thoracoscopic lung surgery [19]. The fundamental mechanism behind a prolonged postoperative analgesia in LB compared to SB is the unique drug delivery system of LB [20]. Although SB is a commonly used local anesthetic with a low rate of significant adverse events, it rapidly diffuses and typically only lasts between 8 and 10 h. LB is derived from bupivacaine hydrochloride encapsulated within multiple, nonconcentric lipid bi‐layers, which allows rapid absorption of bupivacaine and prolonged delivery to the target site [21]. The analgesic effect of SB may have begun to decline early post‐surgery, while LB provides a sustained block that may cover the peak period of pain. This explains the observations of reduced NRS scores at 24 and 36 h postoperatively and prolonged time to first request for analgesia in the LB group compared to the SB group. Similar results have been obtained from the study of Wang et al. [12] which compared use of LB to ropivacaine in TPVB for managing postoperative pain following VATS. Although our study showed similar results as their study, a specific focus on high‐risk analysis for the age group may make a unique contribution to pain relief post VATS. A mean difference of 1.0 has been considered to reflect a minimal clinically important difference (MCID) in NRS scores [22]. However, the percent reduction in pain at meaningful relief remained stable across baseline pain levels, suggesting patients assess meaningful relief in relative rather than absolute terms [23]. Although the mean difference (0.6) of NRS score in the LB group was below the MCID, this group presented a higher QoR‐15 score at 24 h after surgery with less cumulative sufentanil consumption from 24 to 72 h after surgery compared to the SB group, indicating functional improvements offered by the use of LB in TPVB, such as earlier mobilization and improved respiratory function. In a recent study reported by Yang et al. [24], researchers found higher QoR‐15 scores 24 h after surgery with longer time to the first analgesia request in the LB group than in the SB group, which were consistent with our results. However, unlike our results of less cumulative sufentanil consumption from 24 to 72 h post‐surgery in the LB group than in the SB group, they did not find a significant difference in opioid consumption between two groups. Two injection sites of local anesthetic in their study and only older patients included in our study may be taken into account when interpreting the different results.

Effective pain control has been identified as an approach to mitigate central sensitization, which plays an important role in developing chronic post‐surgical pain [25]. Prolonged release of LB provides adequate and sustained peripheral nerve block, which can prevent or reduce the transmission of nociceptive signals caused by surgical trauma to the central nervous system, thereby reducing central sensitization to prevent chronic post‐surgical pain. Due to age‐related decline in physiological reserve and loss of efficiency of homeostatic mechanisms in older patients, the use of analgesic drugs for this population group should take into account opioid savings [26]. The combined multimodal and opioid‐sparing strategy is particularly important for older patients to reduce the risk of opioid‐related side effects such as respiratory depression, nausea and vomiting, somnolence, and delirium [27]. The use of opioids is highly pertinent to the development of postoperative delirium [28]. TPVB effectively blocked the neural afferents, reduced postoperative acute pain, opioid consumption, and neurocognitive dysfunction, thus alleviating the incidence of postoperative delirium in geriatric patients undergoing pulmonary resection [29]. The inclusion of LB in TPVB may lead to notable decreases in opioid requirement in all postoperative days studied with a trend toward decreased delirium rates as well [30]. As shown in our results, the LB group required significantly less cumulative sufentanil between 24 and 72 h, but the incidence of postoperative delirium did not decrease significantly compared to the SB group, likely due to small sample size and fewer elderly patients involved.

The QoR‐15 shows excellent reliability and clinical feasibility to assess improved recovery metrics during enhanced recovery after surgery (ERAS) protocols [31]. Improved early recovery may prevent the incidence of postoperative complications and development of chronic post‐surgical pain following elective surgeries [32]. The administration of LB in TPVB exhibited an improved QoR and decreased opioid requirements without an increase in the incidence of adverse events, which may foster the adoption of ERAS protocols tailored for VATS. A mean difference of 8.0 was considered to represent a clinically important improvement or deterioration in QoR‐15 scores [33]. In this study, the SB group exhibited a mean difference of 14.7 in clinically important deterioration and the LB group exhibited a mean difference of 8.2 in clinically important deterioration, indicating an improved early recovery in the LB group compared to the SB group. Aligned with recovery metrics in our study, previous studies provided evidence of LB in regional anesthesia as part of ERAS protocols for thoracoscopic surgery [34, 35].

The study has several limitations. First, the single‐center design with a relatively small sample size may constrain the generalizability of the role of this agent in thoracic regional anesthesia. Possible multi‐center trials with larger‐scale populations would highlight the applicability of this thoracic regional anesthesia to broader clinical settings. Second, the observed mean difference of the NRS score in the LB group below the MCID may limit the ability to realize the full potential benefits of the use of LB in TPVB for older patients during VATS, which also creates a need for validation using a 100‐mm visual analog scale or in a multi‐center trial with larger‐scale populations. Third, the substantial cost disparity between LB and SB necessitates formal health‐economic evaluations and comparative cost‐utility analyses to determine economic feasibility in resource‐limited settings, despite potential hospitalization cost reductions from decreased opioid use. Fourth, no long‐term recovery outcome was reported in this study, which required a longer follow‐up assessment (3 or 6 months) to test the potential of this agent to reduce the risk of chronic postsurgical pain. Fifth, the attending anesthesiologist not being blinded to the trial grouping may affect the results of the quality of anesthesia recovery and degree of nausea and vomiting, as anesthesiologists may implicitly bias their predictions toward study medication.

In conclusion, our results demonstrate use of LB in ultrasound‐guided TPVB could offer prolonged analgesia at 24 and 36 h postoperatively without adding adverse events compared to SB, translating to improved recovery metrics and opioid savings for older patients undergoing VATS. This study coincides well with the unique value for the role of LB within ERAS protocols and provides evidence for optimizing multimodal analgesic strategies, which may address the paucity of evidence on this strategy in thoracic regional anesthesia for this specific patient population. Considering relatively higher costs of LB compared to SB, its cost efficiency should be further investigated to enhance its availability and feasibility in resource‐limited settings.

## Funding

This work was supported by Taicang Science and Technology Bureau (TC2024JCYL19).

## Ethics Statement

The study protocol complied with the principles of the Declaration of Helsinki and was approved by the Ethics Committee of Taicang First People's Hospital. All participants or legal representatives provided written informed consent.

## Conflicts of Interest

The authors declare no conflicts of interest.

## Data Availability

The data that support the findings of this study are available from the corresponding author upon reasonable request.
